# Mathematical modelling of ICAN-mediated persistent firing in hippocampal neurons

**DOI:** 10.1186/1471-2202-16-S1-P292

**Published:** 2015-12-18

**Authors:** Francesco Giovannini, Motoharu Yoshida, Laure Buhry

**Affiliations:** 1 Neurosys Team, INRIA, LORIA UMR 7503, CNRS, Université de Lorraine, Villers-lès-Nancy, F-54600, France; 2 Faculty of Psychology, Mercator Research Group - Structure of Memory, Ruhr-University, Bochum, 44801, Germany

## 

Persistent neural activity has been the focus of neuroscientific research since it was first associated with complex cognitive behaviours. In particular, persistent firing has long been thought to be the neural mechanisms underlying short-term memory encoding and storage [1]. This activity is often elicited by short transient stimuli that have to be retained in memory for long delay periods, in the order of several seconds, after the original stimulus disappeared. In this scenario, the brain stores information for future execution of action depending on that information.

Persistent activity elicited in a recurrent network comprising strong excitation in the local circuit has been extensively described in the literature (for a review see [2]). However, recent findings have shown that memory can also be encoded in brain regions which do not display such recurrent connection topology, including the CA1 hippocampal area [3]. Emerging studies are pointing towards intrinsic neural mechanisms independent of synaptic connections, as a complementary mechanism for the maintenance of persistent activity in the hippocampus [4]. These include various cytoplasmic currents flowing through the membrane, characterised by slow ion channel kinetics, and particular neurotransmitter modulation. Our work investigates persistent firing activity in networks of hippocampal neurons, elicited by leveraging intrinsic currents rather than network dynamics.

Building on previous findings [5], we model a hippocampal pyramidal neuron using the Hodgkin-Huxley model, with low-threshold Ca^2+ ^currents governing the inward flow of calcium ions in the cell membrane. The intracellular calcium concentration mediates the opening of calcium-activated non-specific (CAN) ion channels [6], which causes an increase in the ionic flow through the cell membrane. Therefore the CAN channels equip the neuron with an after-spike depolarisation mechanism, which enables it to emit action potentials in the absence of external stimulation. Given a transient 250*ms *200*pA *current injection, the neuron is capable of eliciting and maintaining persistent activity with a firing rate of 6*Hz *for long delay periods (*~30s*). Moreover, this behaviour is in accord with that displayed in neural recordings of hippocampal slice preparations [4]. Connecting these persistent firing neurons in a network comprising strong local excitation yields a wide range of behaviours depending on the interaction between CAN and synaptic currents. Indeed, our network model is capable of displaying rhythmic behaviour in the form of short synchronised bursts with intra-burst frequencies of *20-40Hz *and inter-burst frequencies of *3Hz*. These results hint towards a possible mechanism for the generation of memory-related oscillatory activity in the hippocampus.
